# Supplementary figures and images for: Caenorhabditis elegans SEL-5/AAK1 regulates cell migration and cell outgrowth independently of its kinase activity
Source: eLife. 2024 Jul 19;13:e91054. doi: 10.7554/eLife.91054 (PMC11333045; doi:10.7554/eLife.91054)

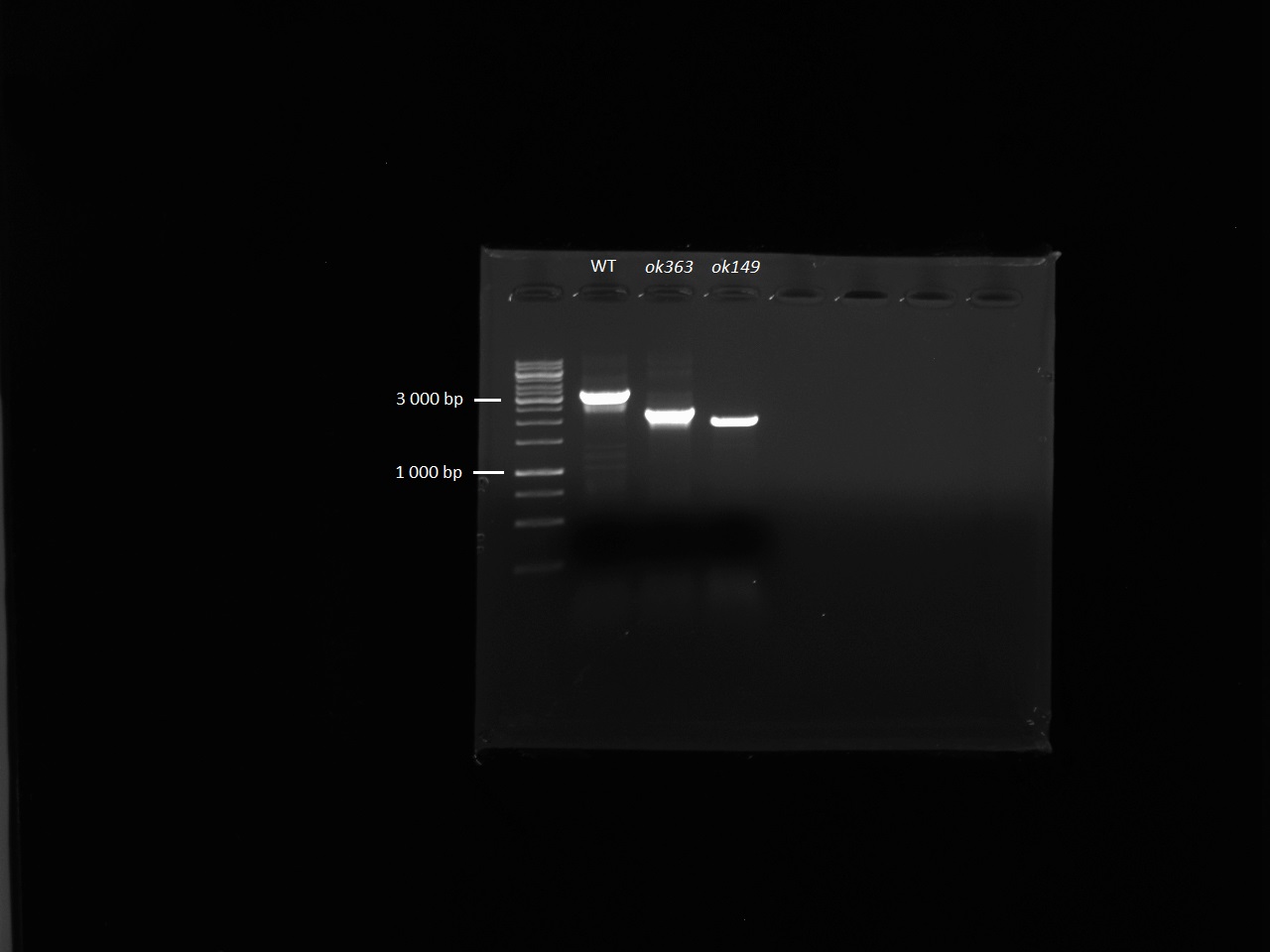

Supplement: Figure 1—source data 1. [file elife-91054-fig1-data1.zip › Figure1SourceData1/Fig1F-raw-labelled.jpg]

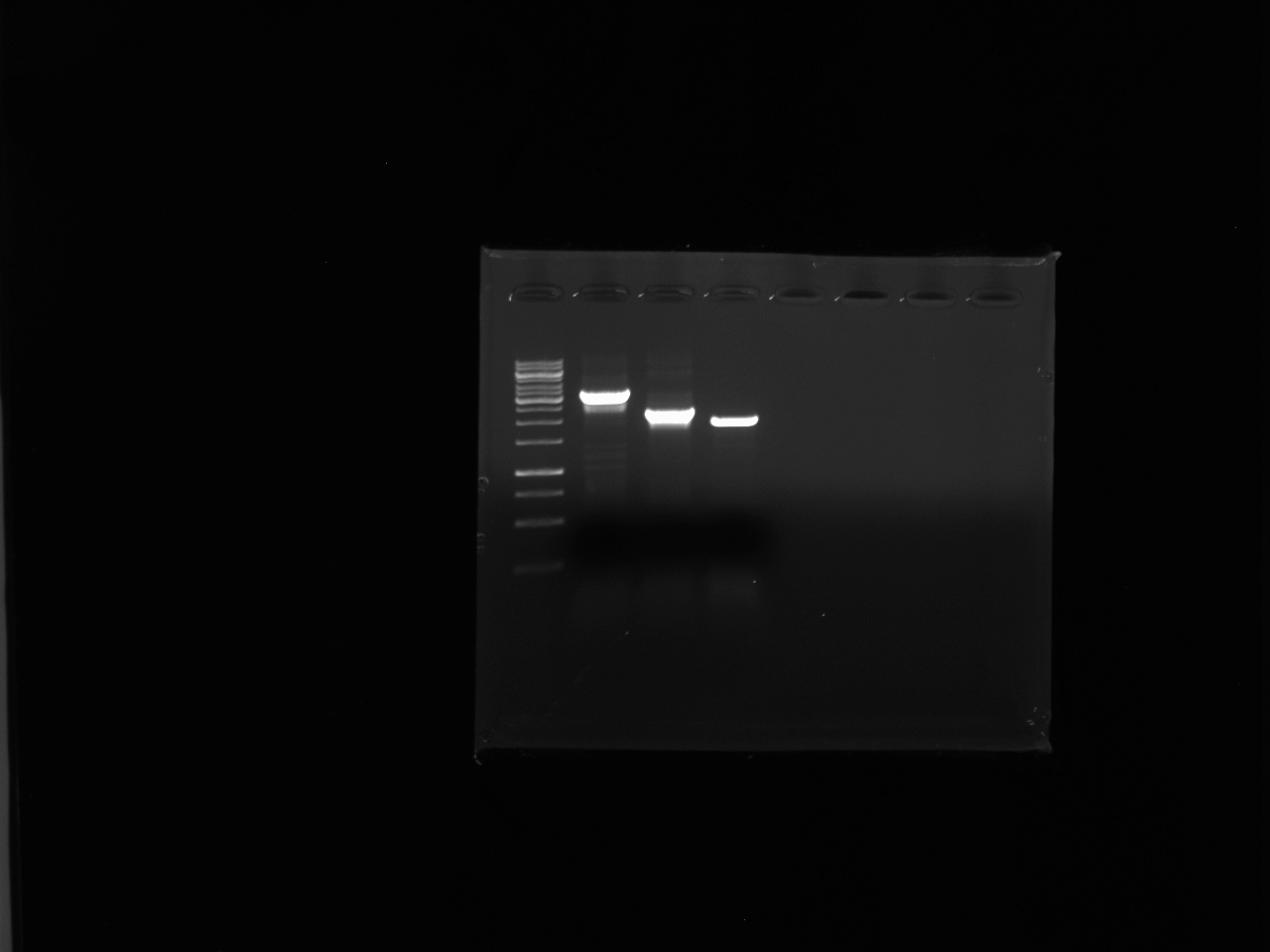

Supplement: Figure 1—source data 1. [file elife-91054-fig1-data1.zip › Figure1SourceData1/Fig1F-raw.jpg]

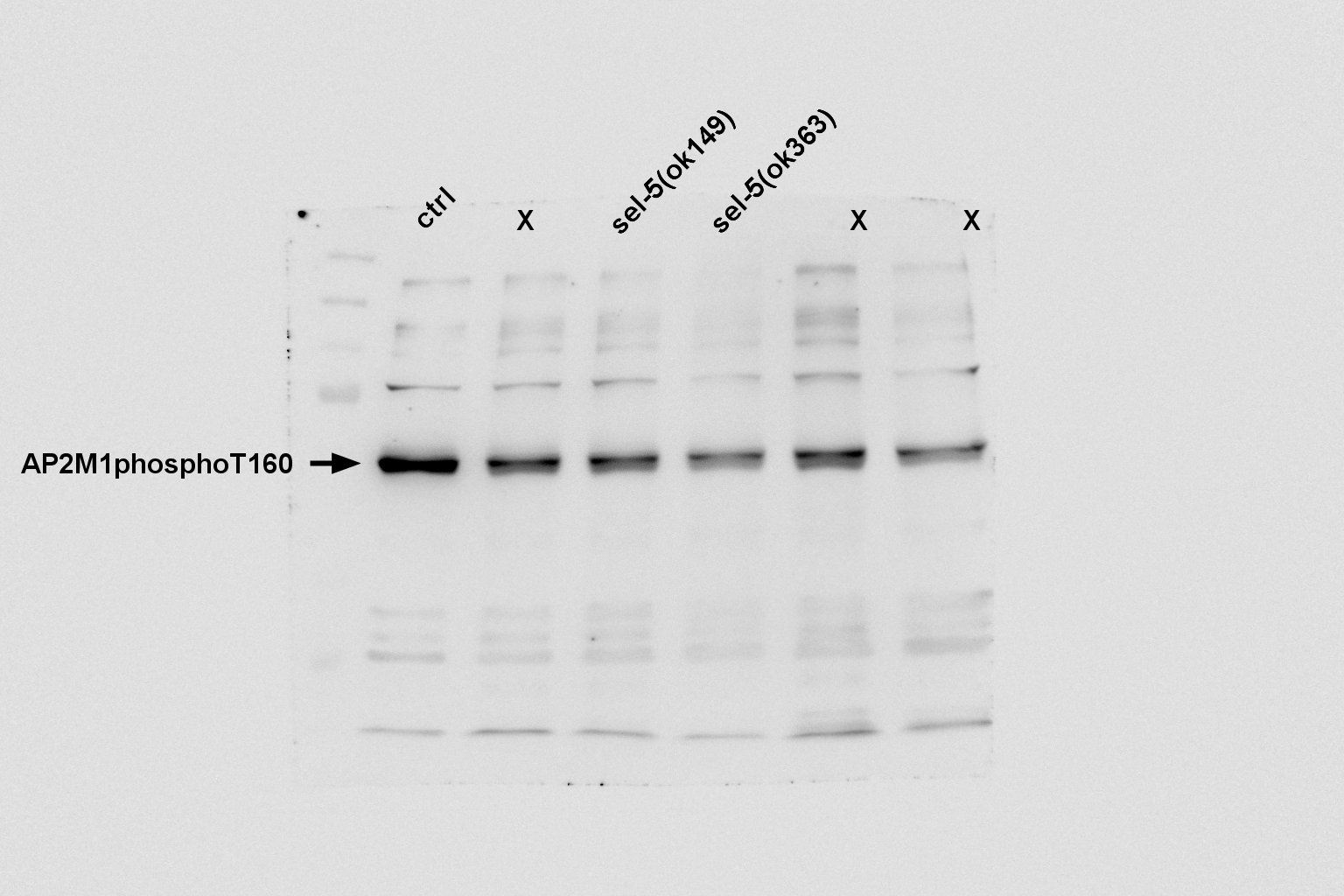

Supplement: Figure 3—source data 1. [file elife-91054-fig3-data1.zip › FIG3A_WB_Sourcedata/FIG3A_01_AP2M1phosphoT160_labelled.jpg]

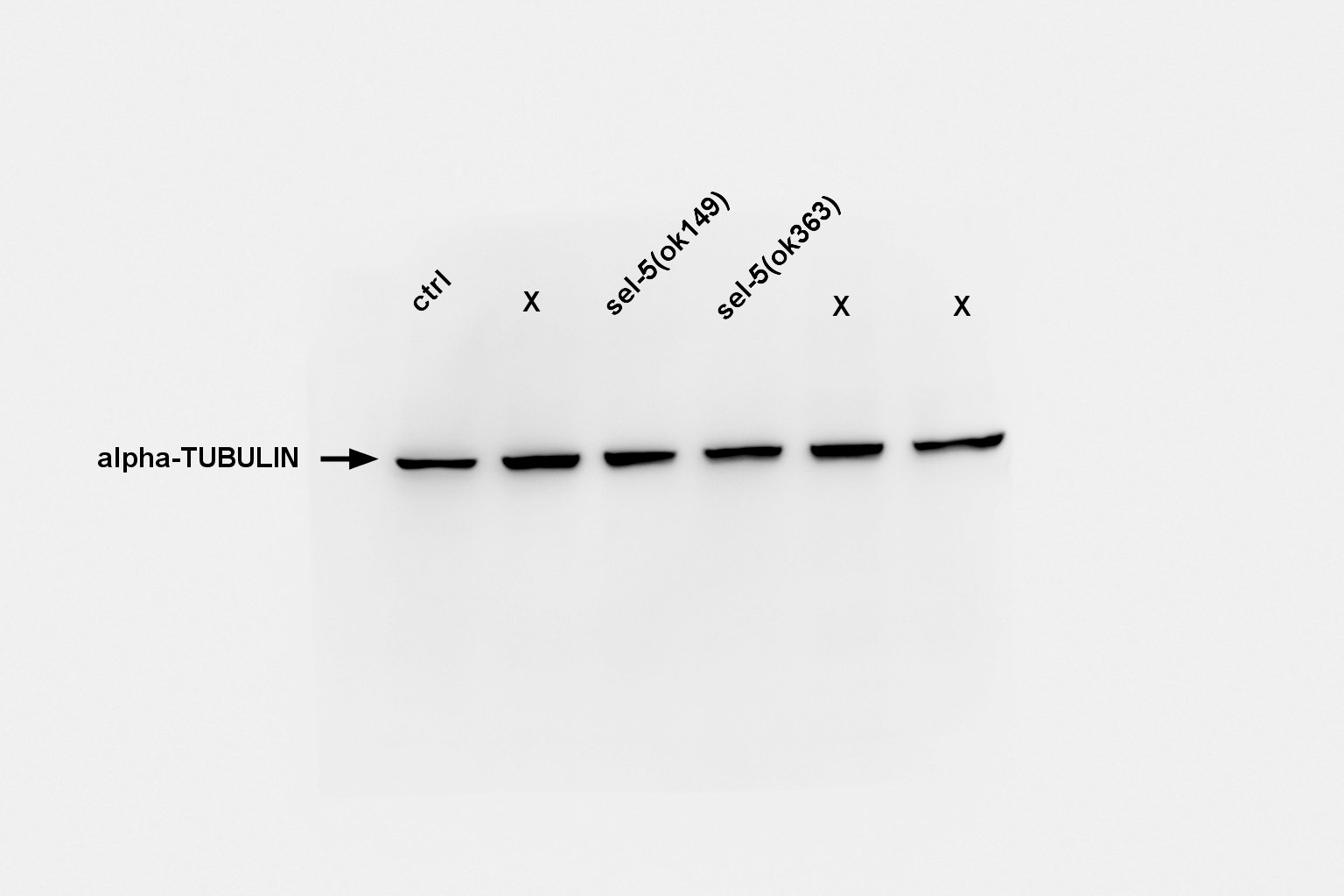

Supplement: Figure 3—source data 1. [file elife-91054-fig3-data1.zip › FIG3A_WB_Sourcedata/FIG3A_01_aTUB_labelled.jpg]

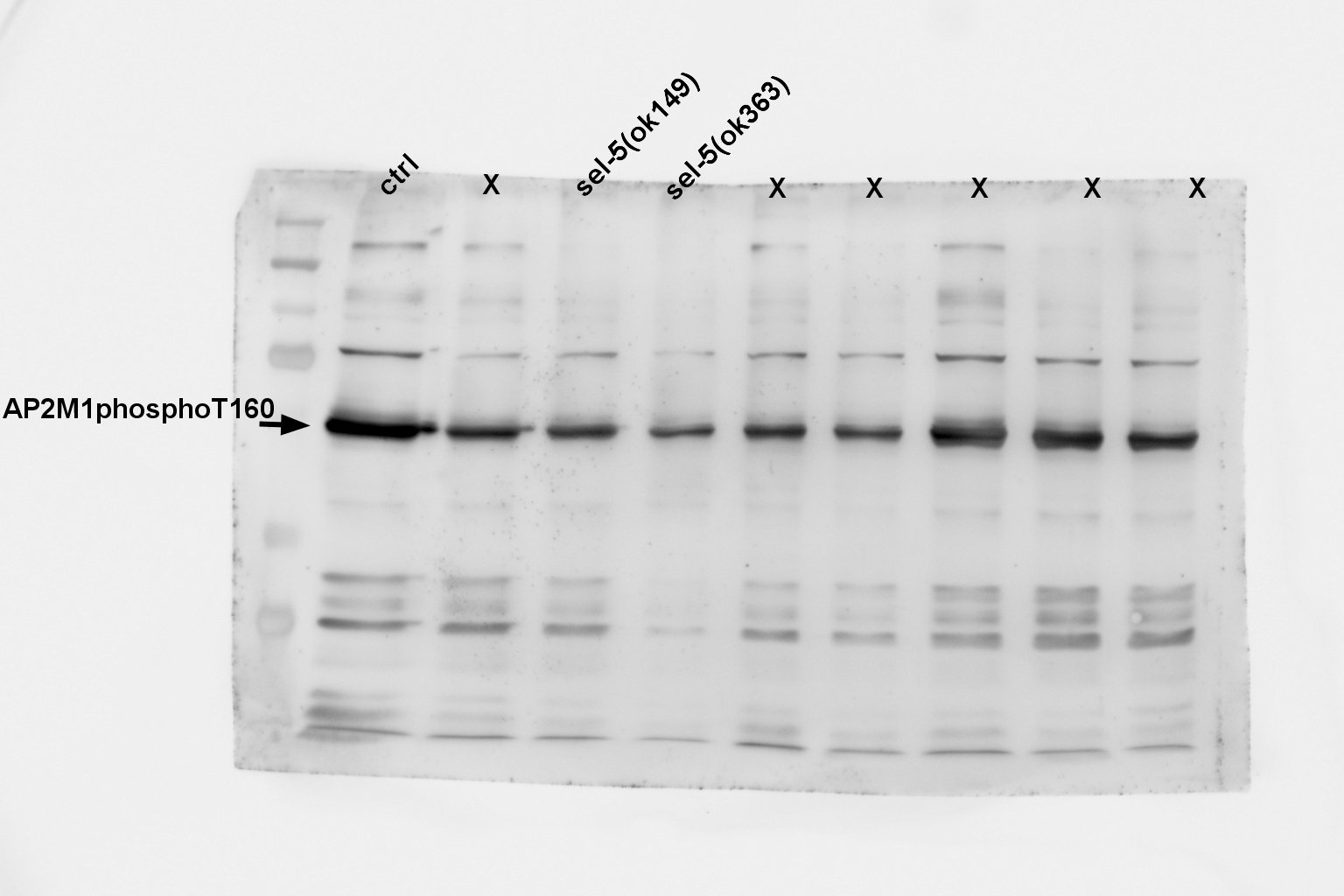

Supplement: Figure 3—source data 1. [file elife-91054-fig3-data1.zip › FIG3A_WB_Sourcedata/FIG3A_02_AP2M1phosphoT160_labelled.jpg]

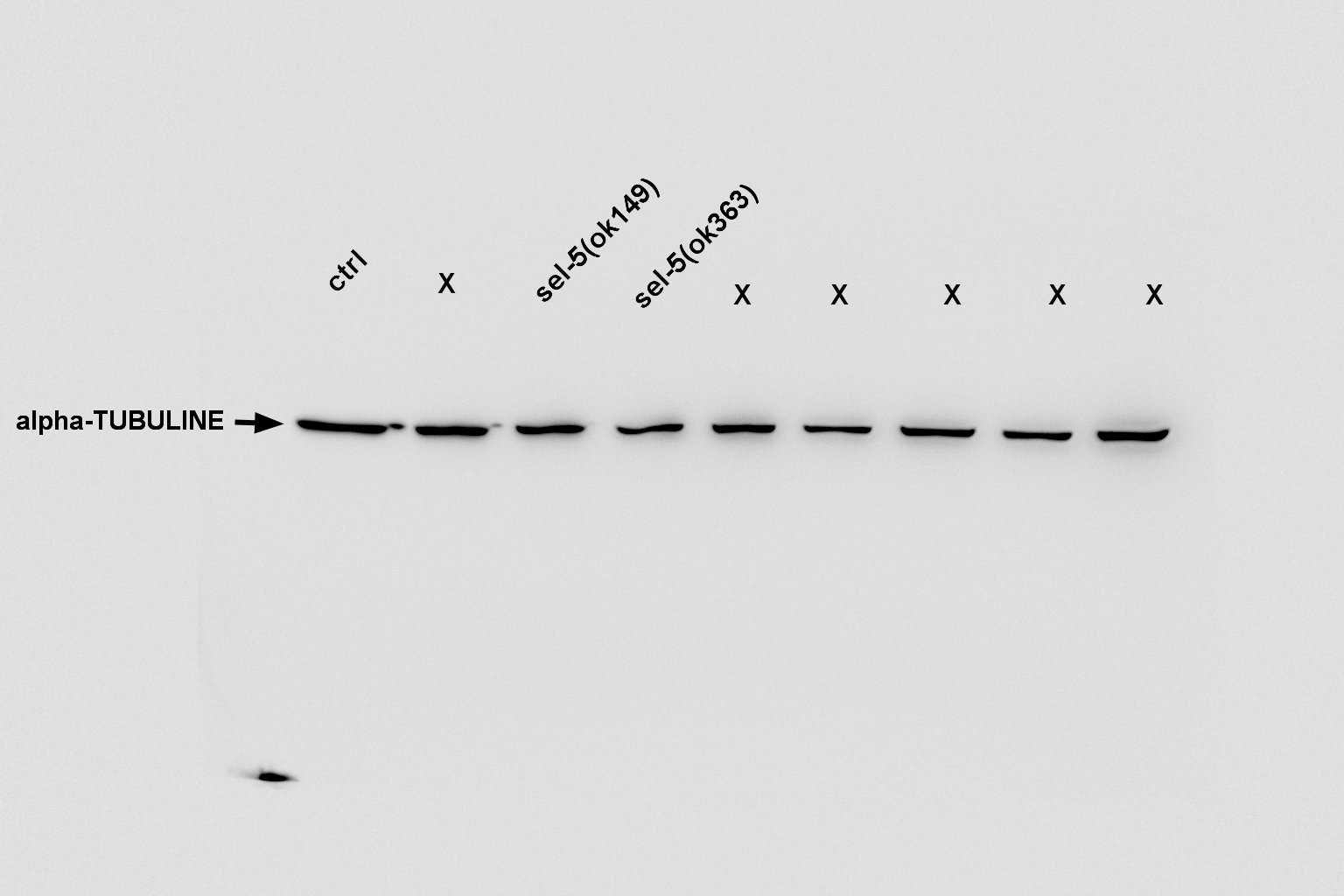

Supplement: Figure 3—source data 1. [file elife-91054-fig3-data1.zip › FIG3A_WB_Sourcedata/FIG3A_02_aTUB_labelled.jpg]

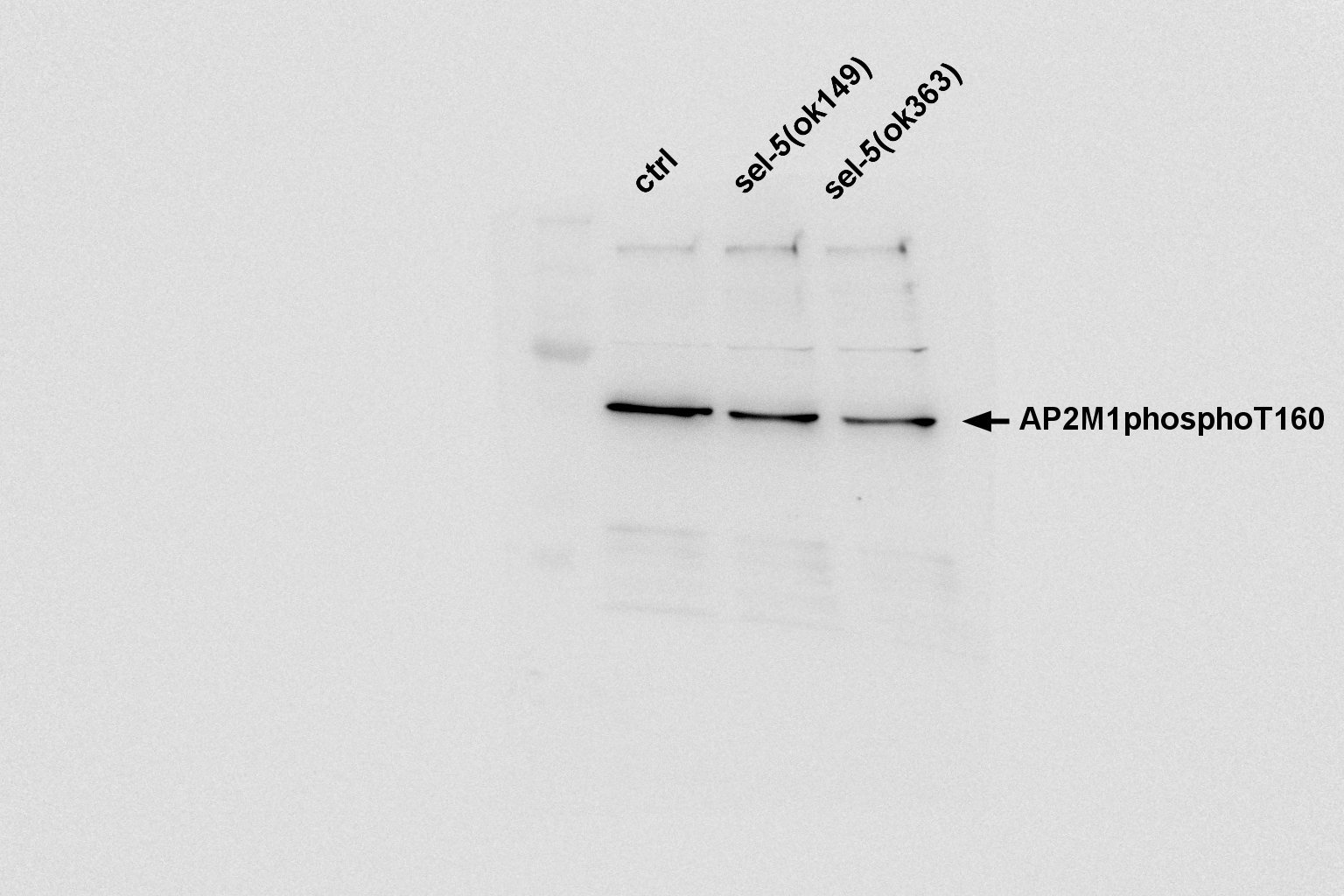

Supplement: Figure 3—source data 1. [file elife-91054-fig3-data1.zip › FIG3A_WB_Sourcedata/FIG3A_03_AP2M1phosphoT160_labelled.jpg]

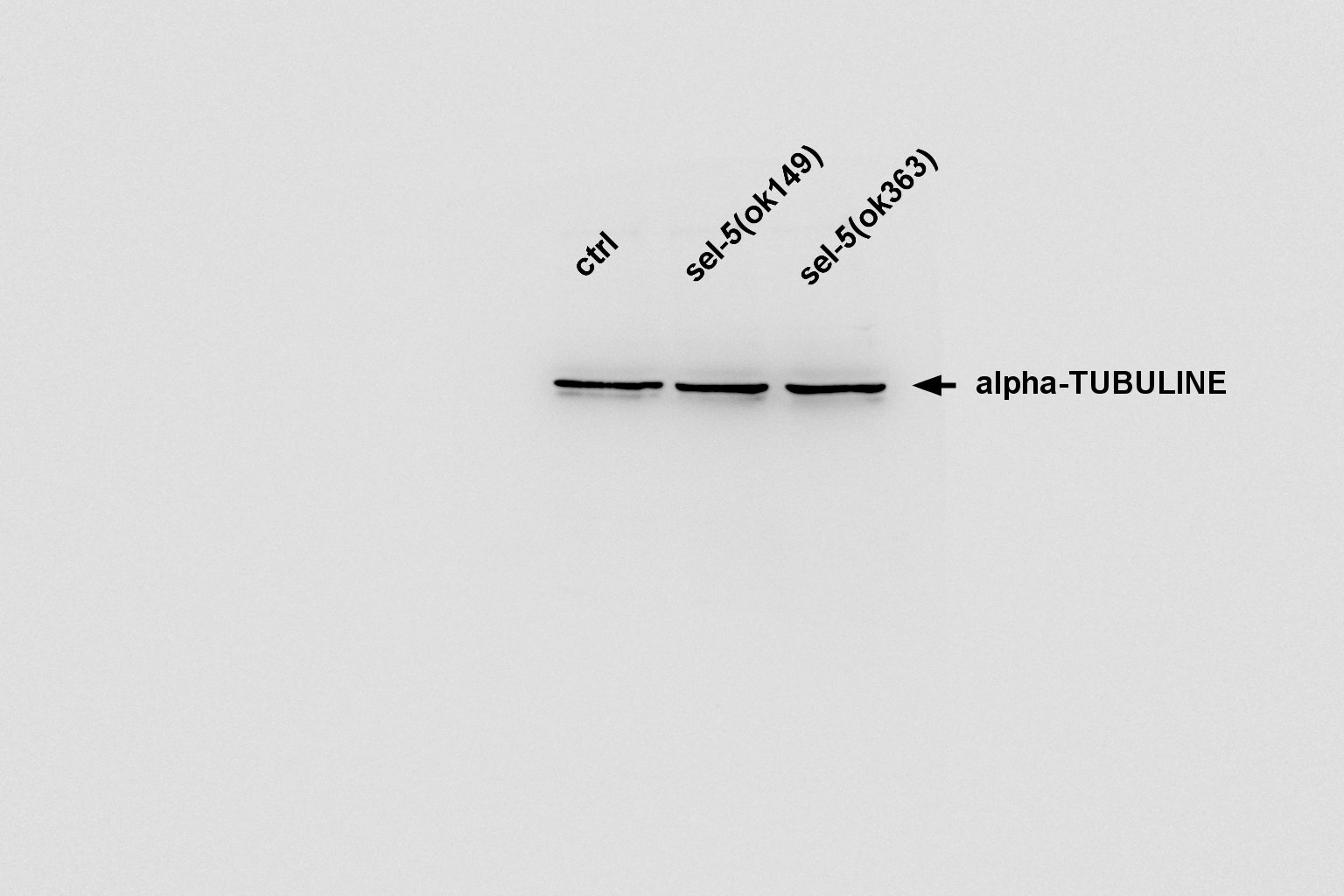

Supplement: Figure 3—source data 1. [file elife-91054-fig3-data1.zip › FIG3A_WB_Sourcedata/FIG3A_03_aTUB_labelled.jpg]

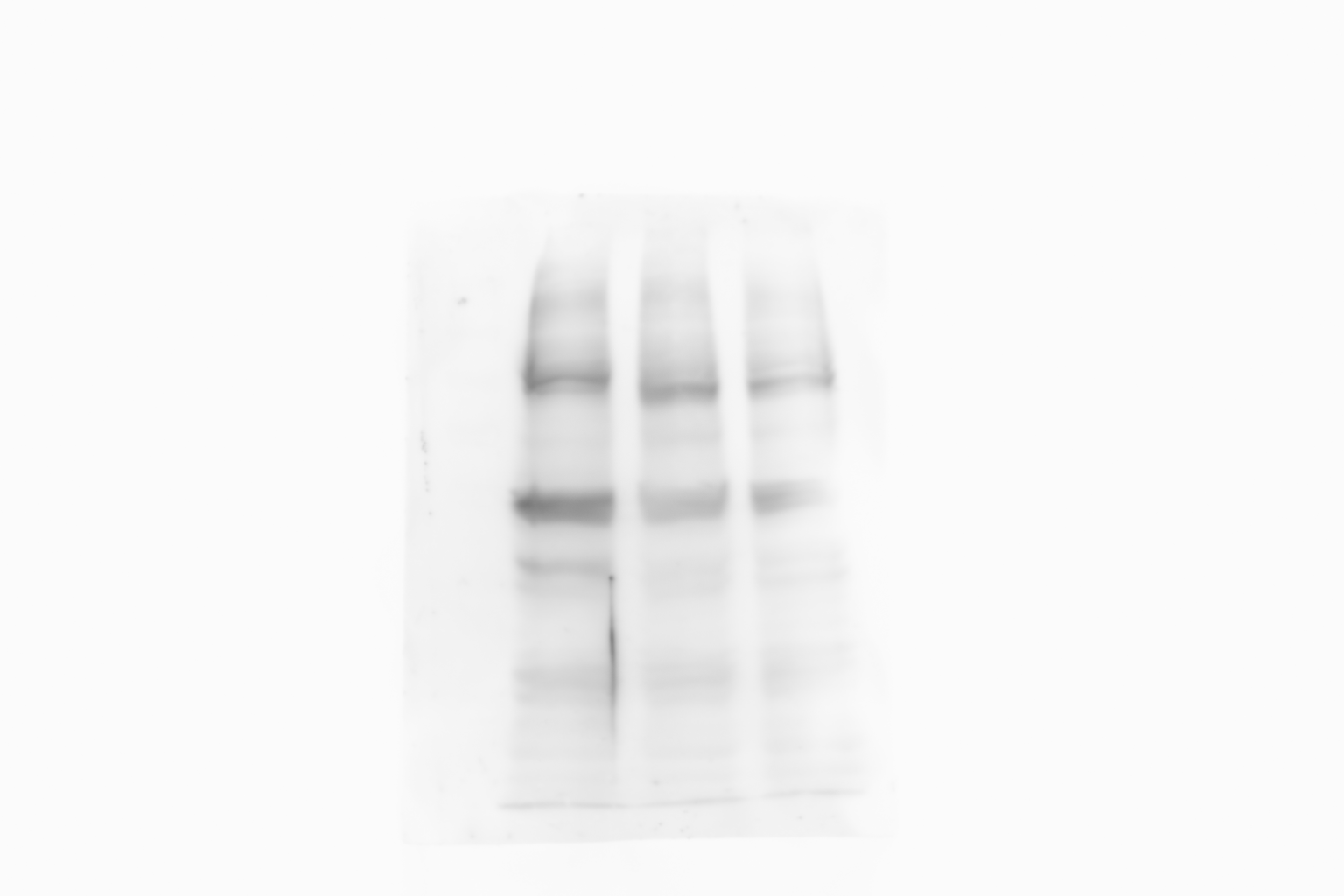

Supplement: Figure 3—source data 1. [file elife-91054-fig3-data1.zip › FIG3A_WB_Sourcedata/FIG3A_04_AP2M1phosphoT160.tif]

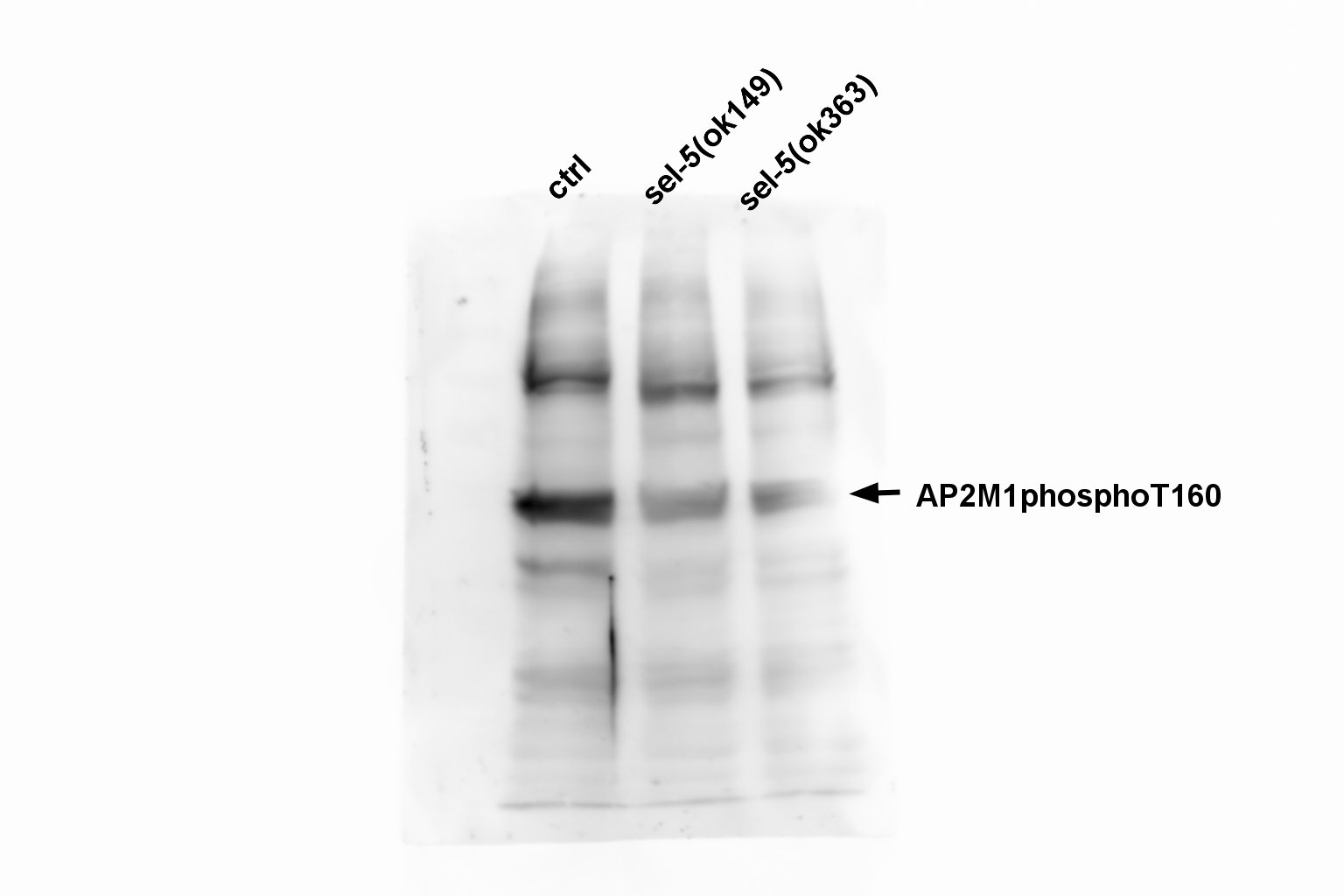

Supplement: Figure 3—source data 1. [file elife-91054-fig3-data1.zip › FIG3A_WB_Sourcedata/FIG3A_04_AP2M1phosphoT160_labelled.jpg]

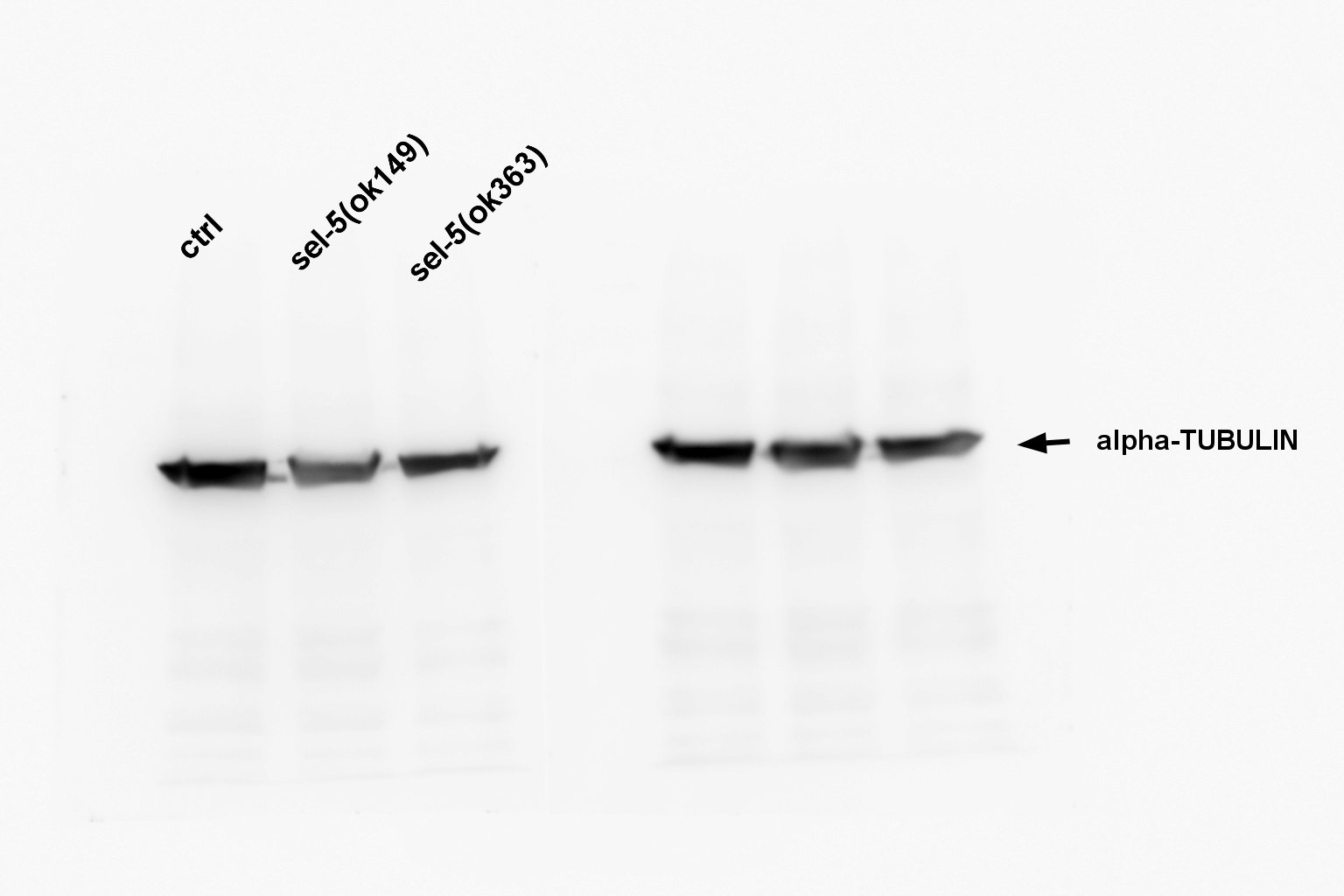

Supplement: Figure 3—source data 1. [file elife-91054-fig3-data1.zip › FIG3A_WB_Sourcedata/FIG3A_04_aTUB_labelled.jpg]

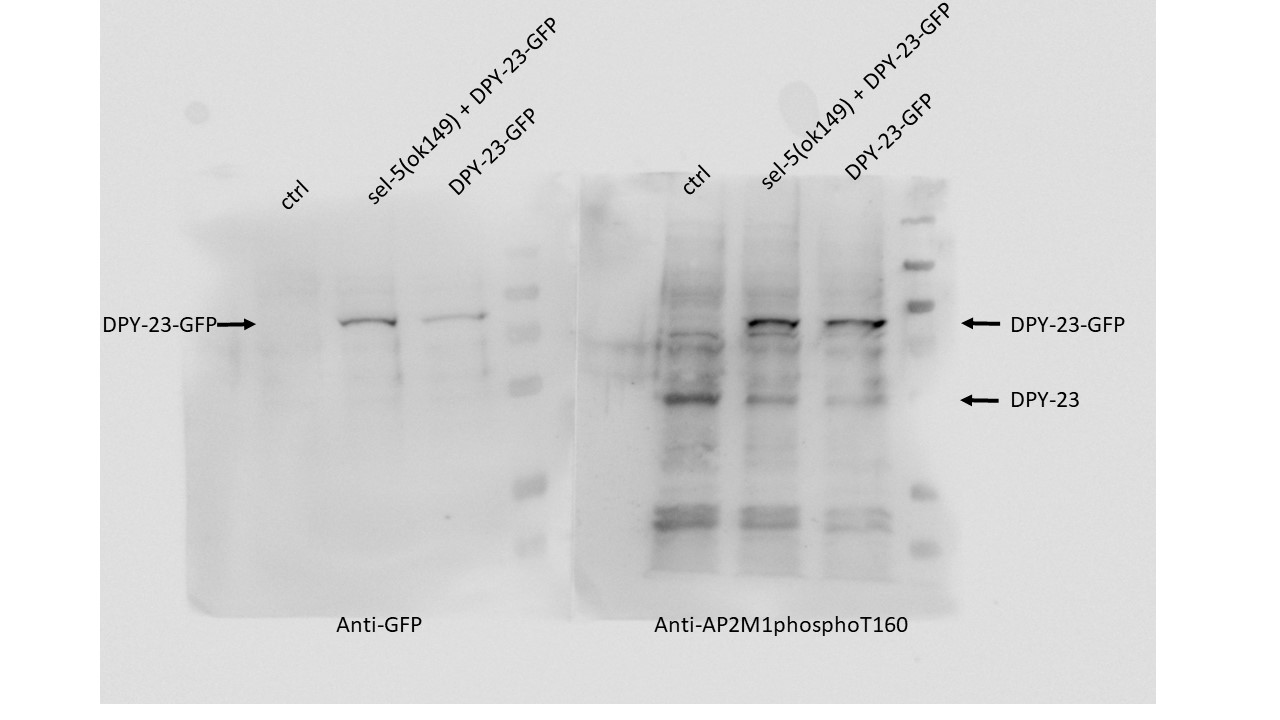

Supplement: Figure 3—source data 1. [file elife-91054-fig3-data1.zip › FIG3B_WB_Sourcedata/FIG3B_01_AP2M1GFP_labelled.jpg]

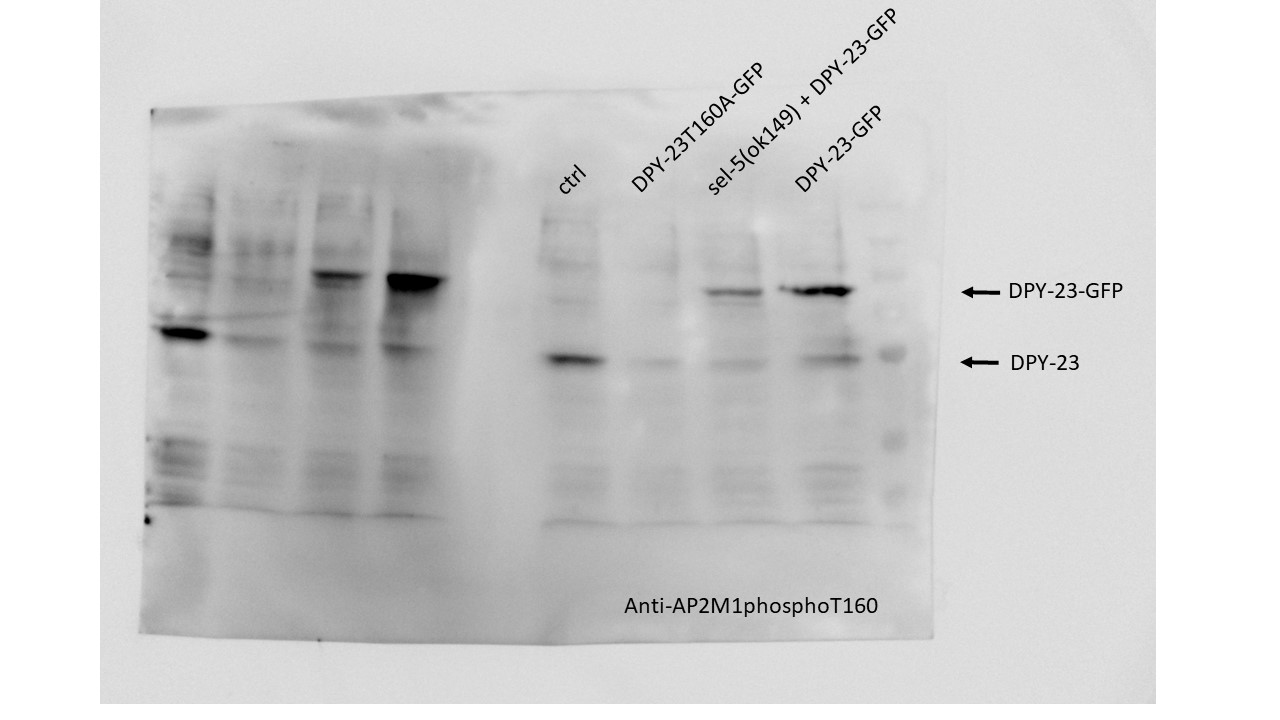

Supplement: Figure 3—source data 1. [file elife-91054-fig3-data1.zip › FIG3B_WB_Sourcedata/FIG3B_02_AP2M1_labelled.jpg]

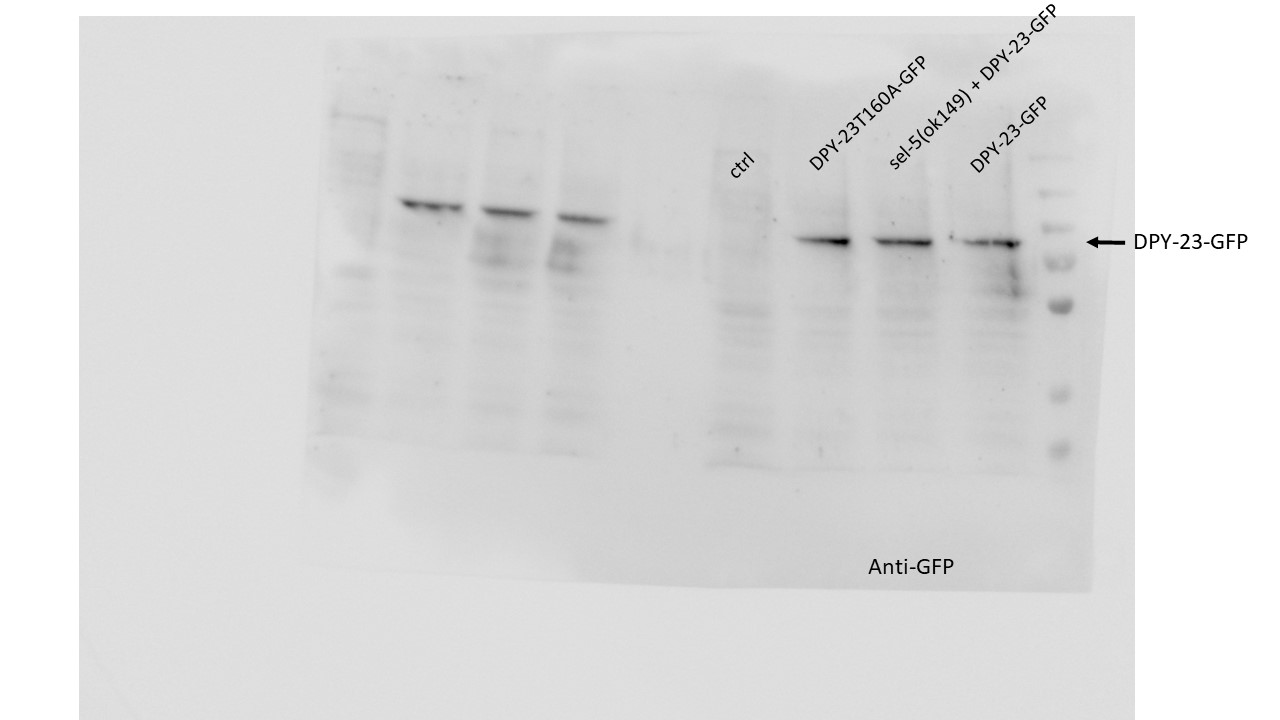

Supplement: Figure 3—source data 1. [file elife-91054-fig3-data1.zip › FIG3B_WB_Sourcedata/FIG3B_02_GFP_labelled.jpg]

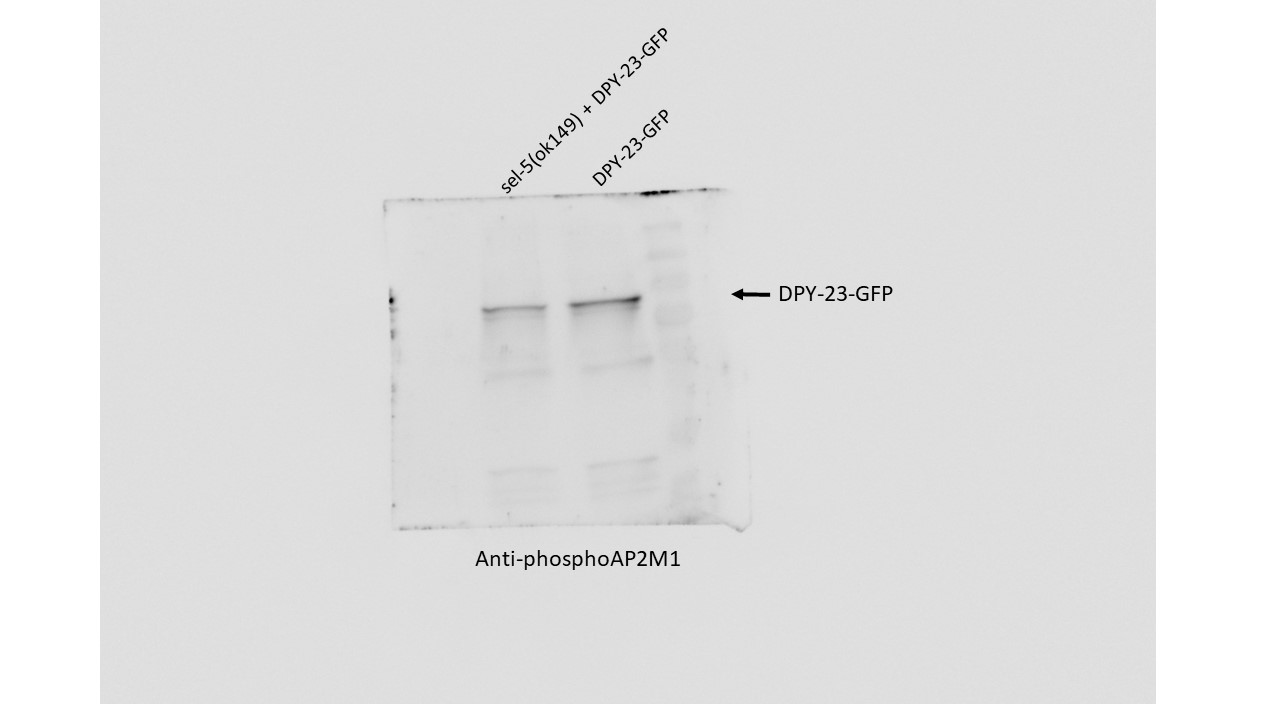

Supplement: Figure 3—source data 1. [file elife-91054-fig3-data1.zip › FIG3B_WB_Sourcedata/FIG3B_03_AP2M1_labelled.jpg]

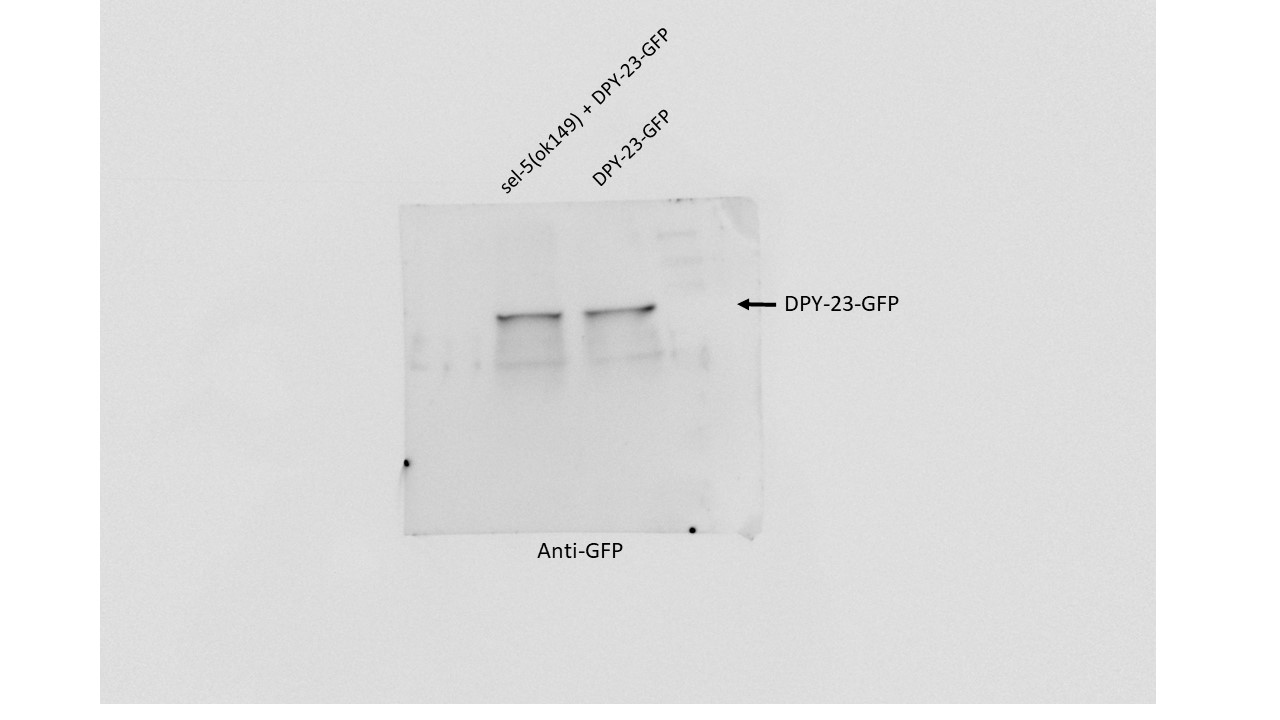

Supplement: Figure 3—source data 1. [file elife-91054-fig3-data1.zip › FIG3B_WB_Sourcedata/FIG3B_03_GFP_labelled.jpg]

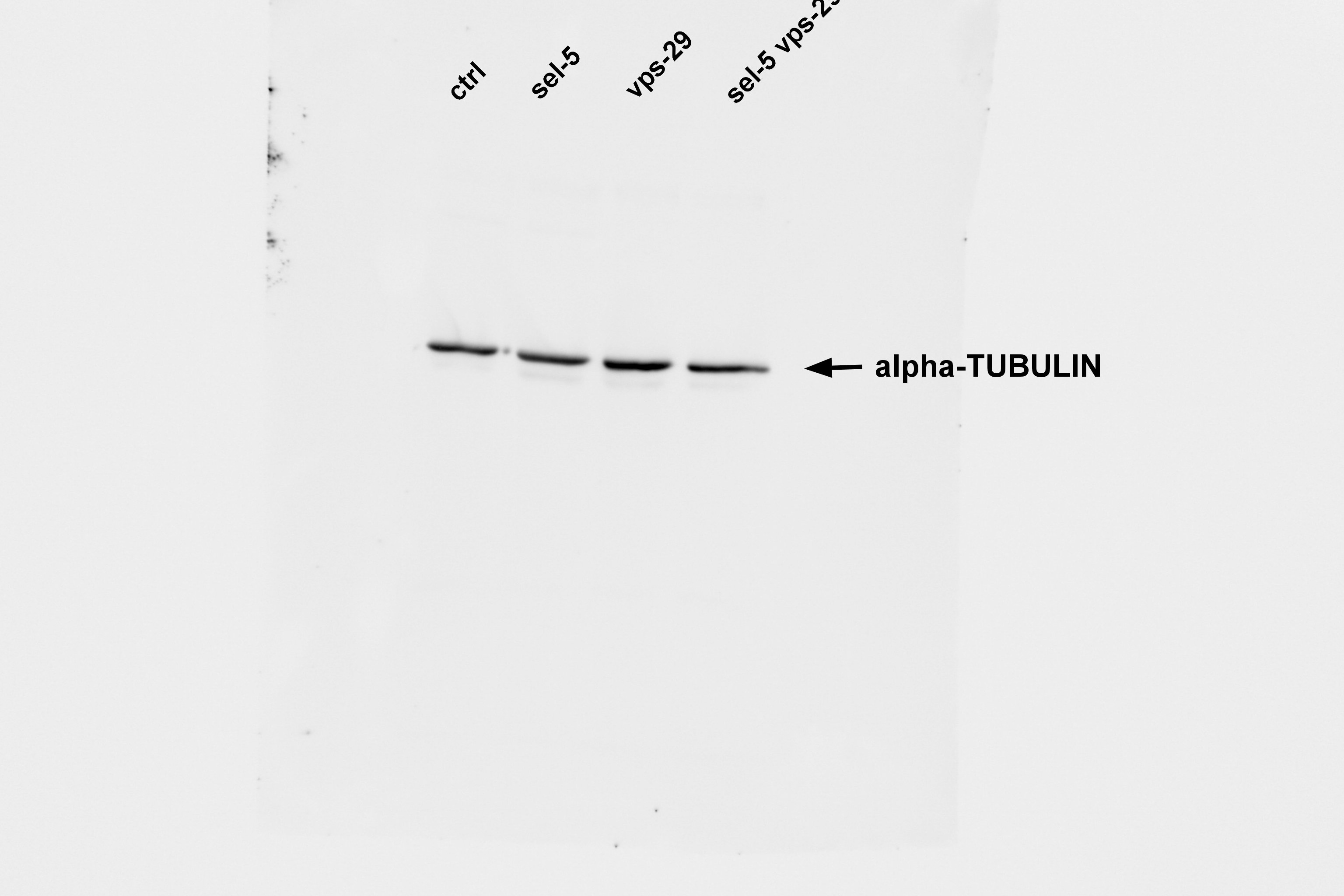

Supplement: Figure 3—source data 1. [file elife-91054-fig3-data1.zip › FIG3D_WB_Sourcedata/FIG3D_01_aTUB_labelled.jpg]

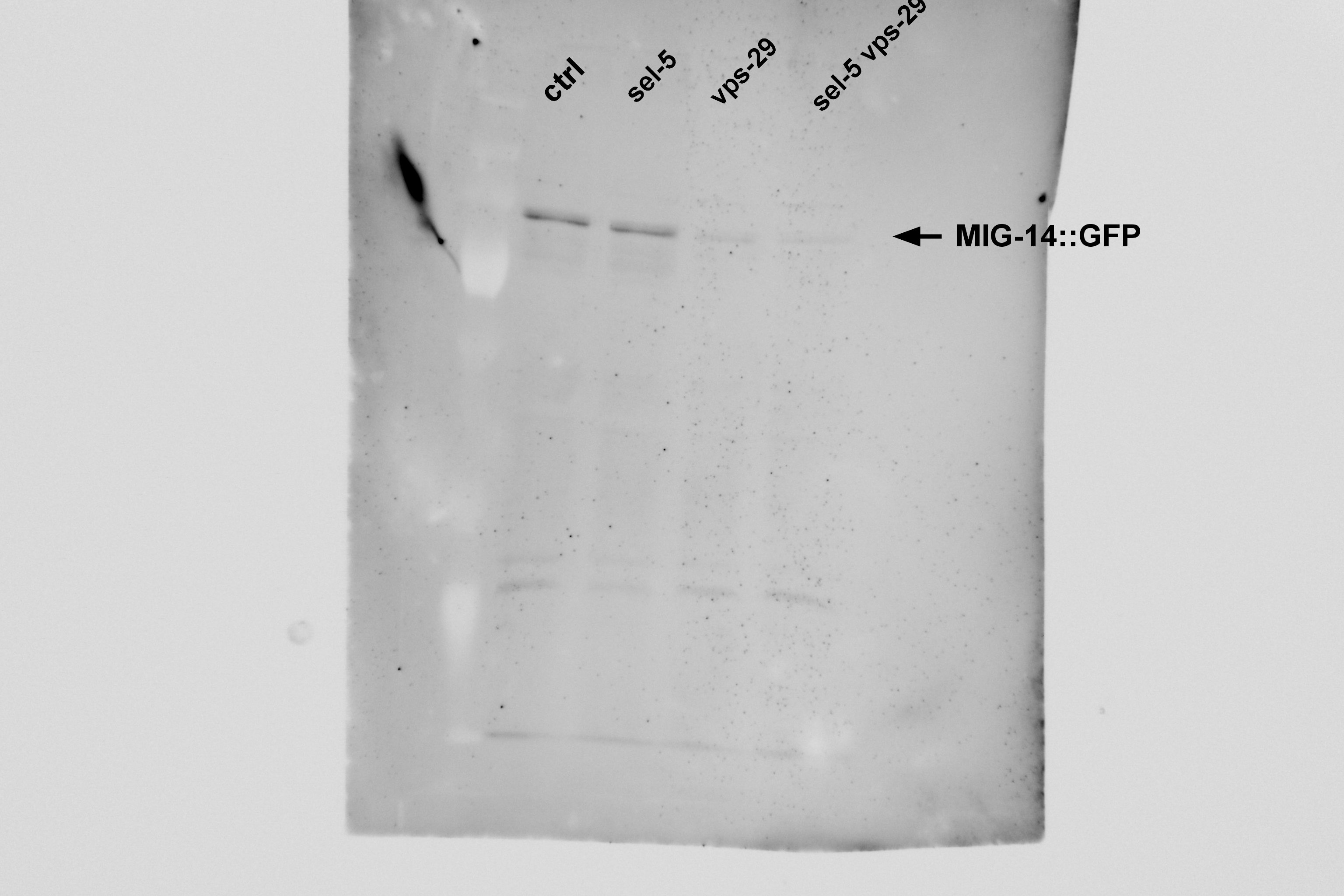

Supplement: Figure 3—source data 1. [file elife-91054-fig3-data1.zip › FIG3D_WB_Sourcedata/FIG3D_01_MIG14GFP_labelled.jpg]

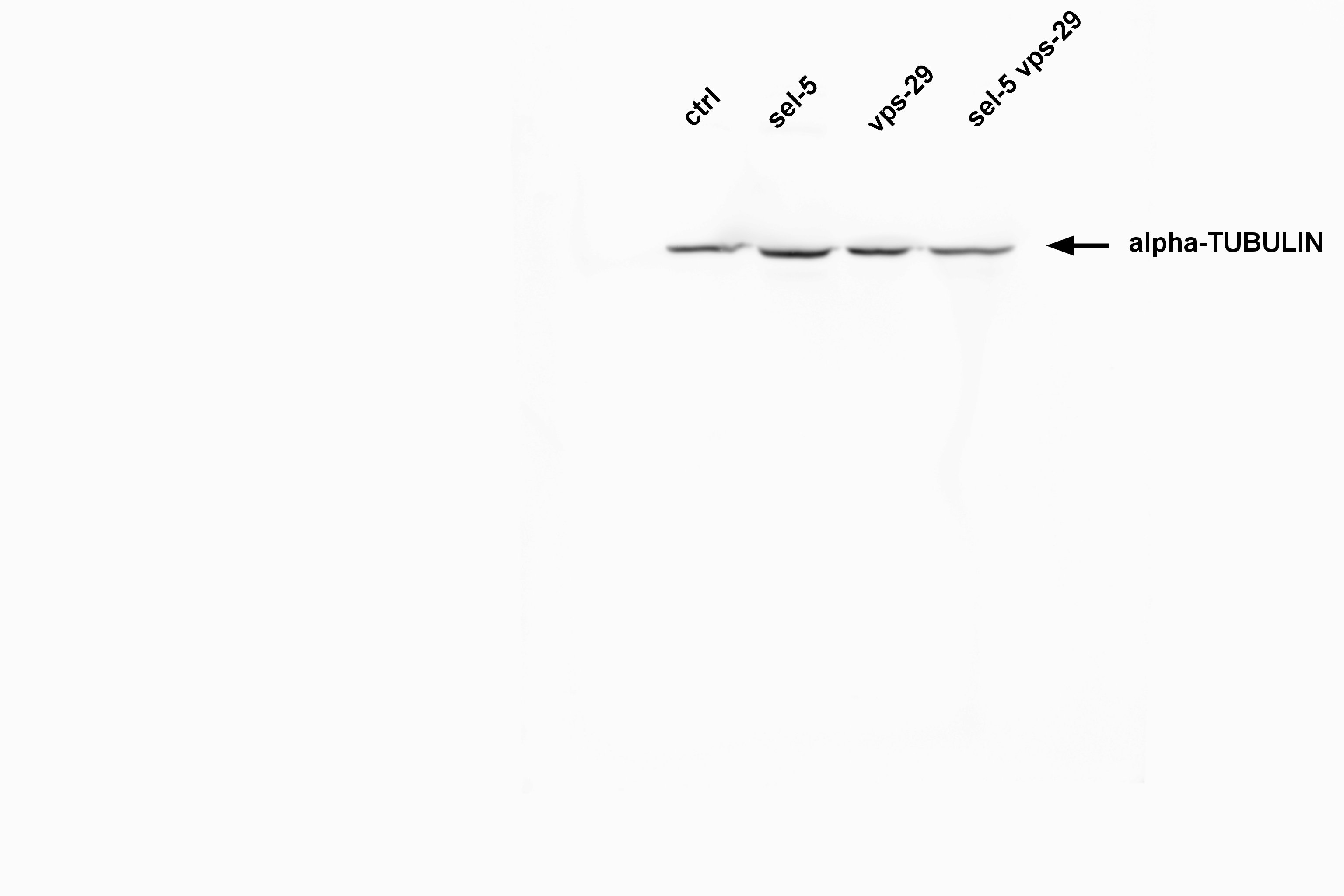

Supplement: Figure 3—source data 1. [file elife-91054-fig3-data1.zip › FIG3D_WB_Sourcedata/FIG3D_02_aTUB_labelled.jpg]

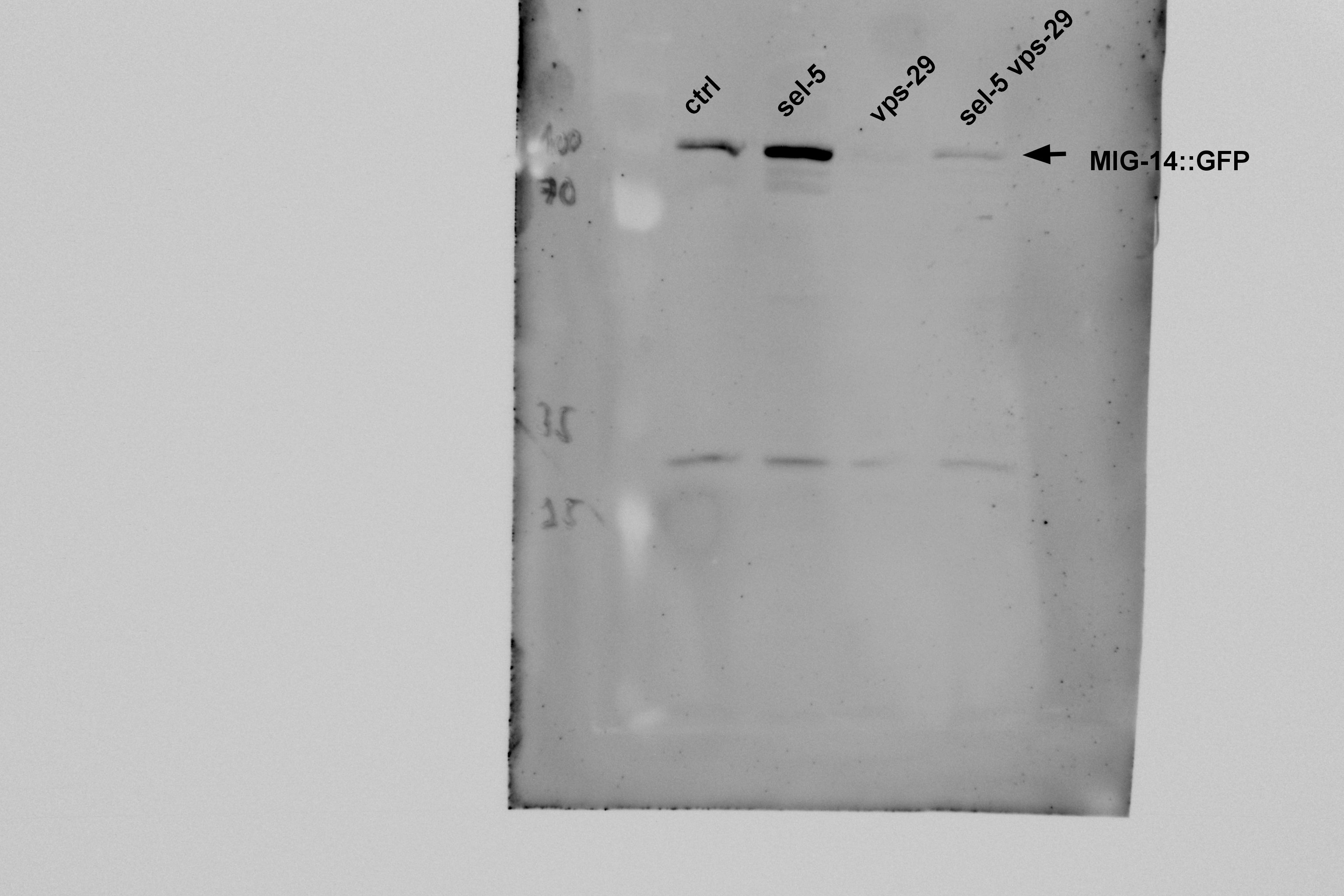

Supplement: Figure 3—source data 1. [file elife-91054-fig3-data1.zip › FIG3D_WB_Sourcedata/FIG3D_02_MIG14GFP_labelled.jpg]

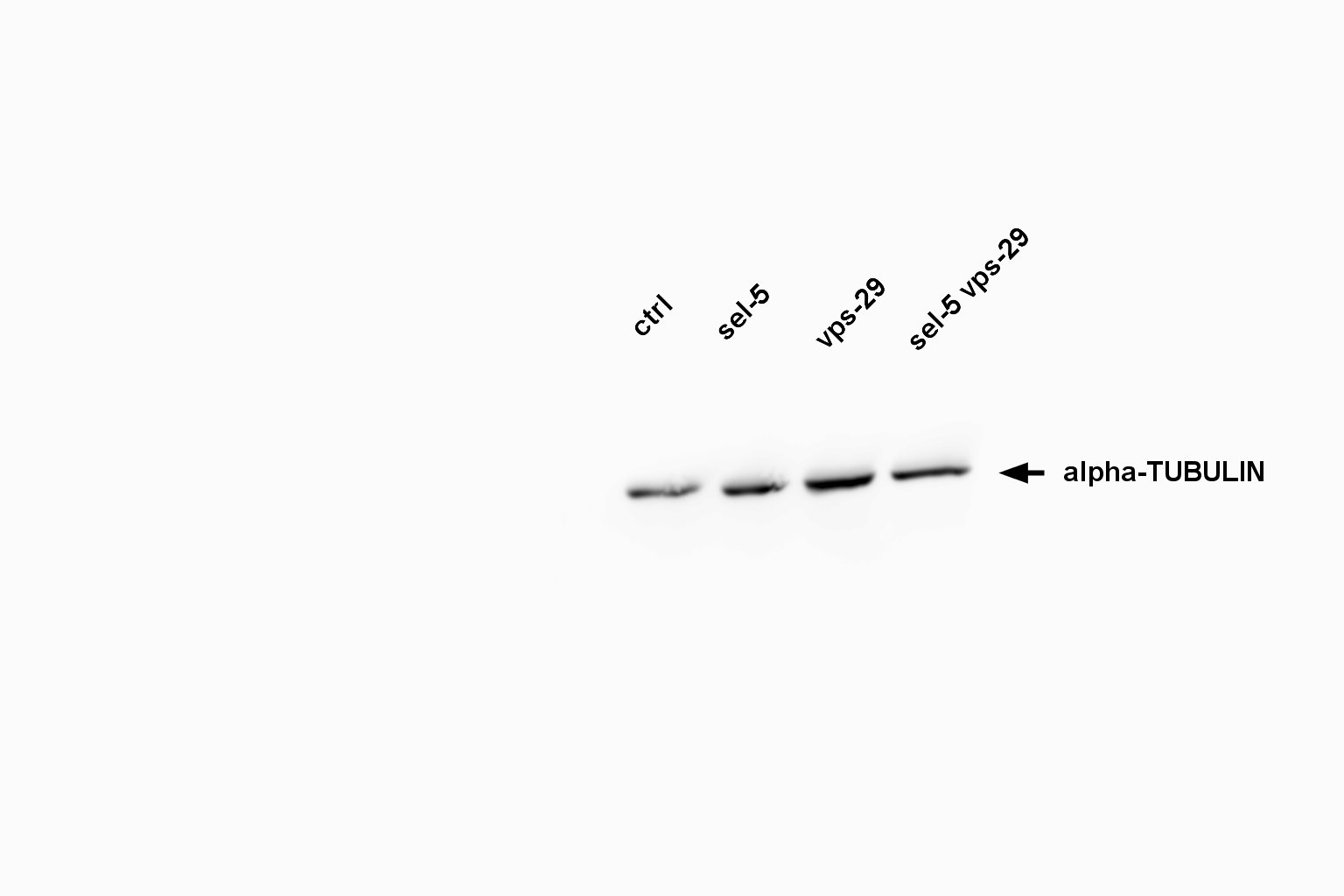

Supplement: Figure 3—source data 1. [file elife-91054-fig3-data1.zip › FIG3D_WB_Sourcedata/FIG3D_03_aTUB_labelled.jpg]

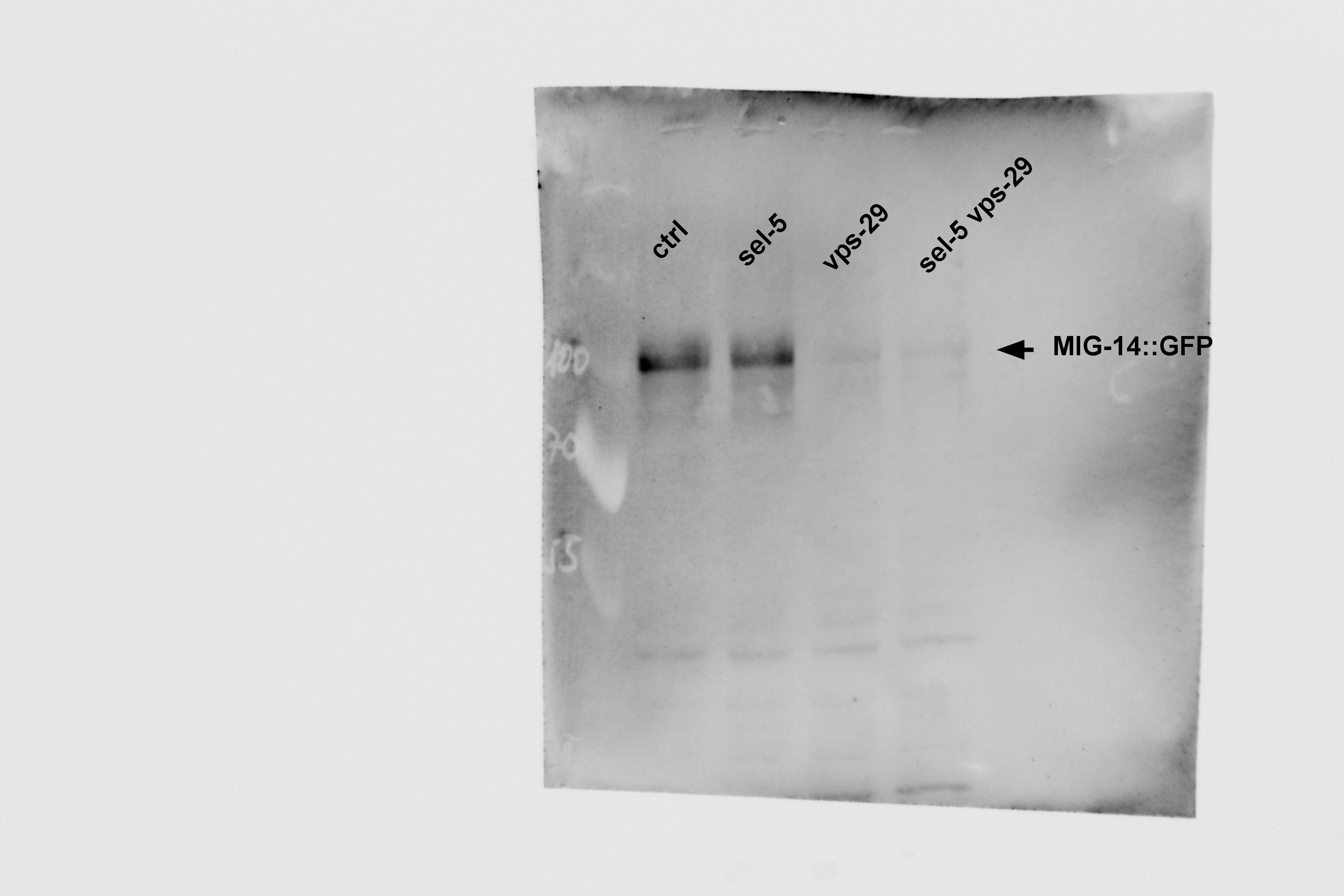

Supplement: Figure 3—source data 1. [file elife-91054-fig3-data1.zip › FIG3D_WB_Sourcedata/FIG3D_03_MIG14GFP_labelled.jpg]

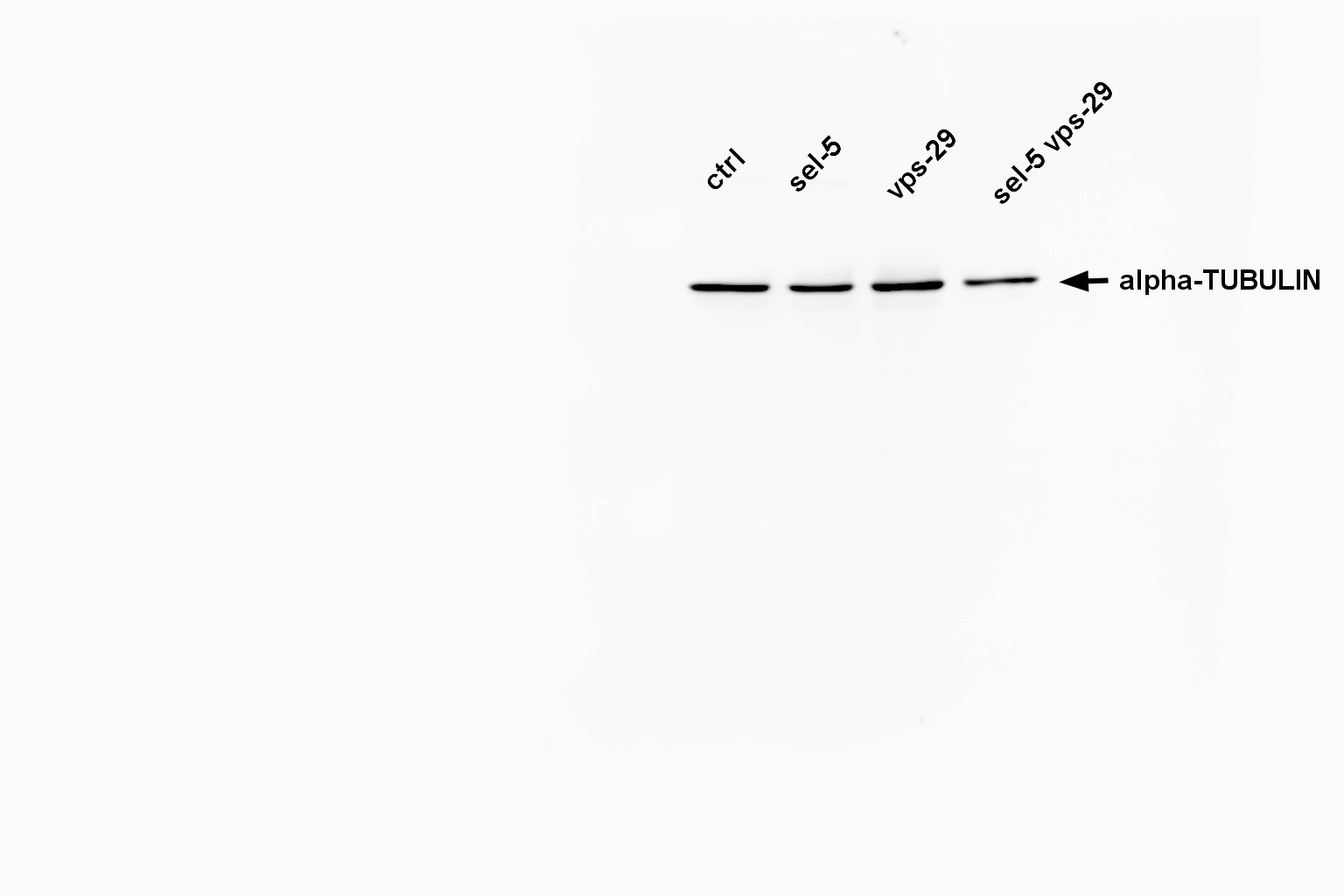

Supplement: Figure 3—source data 1. [file elife-91054-fig3-data1.zip › FIG3D_WB_Sourcedata/FIG3D_04_aTUB_labelled.jpg]

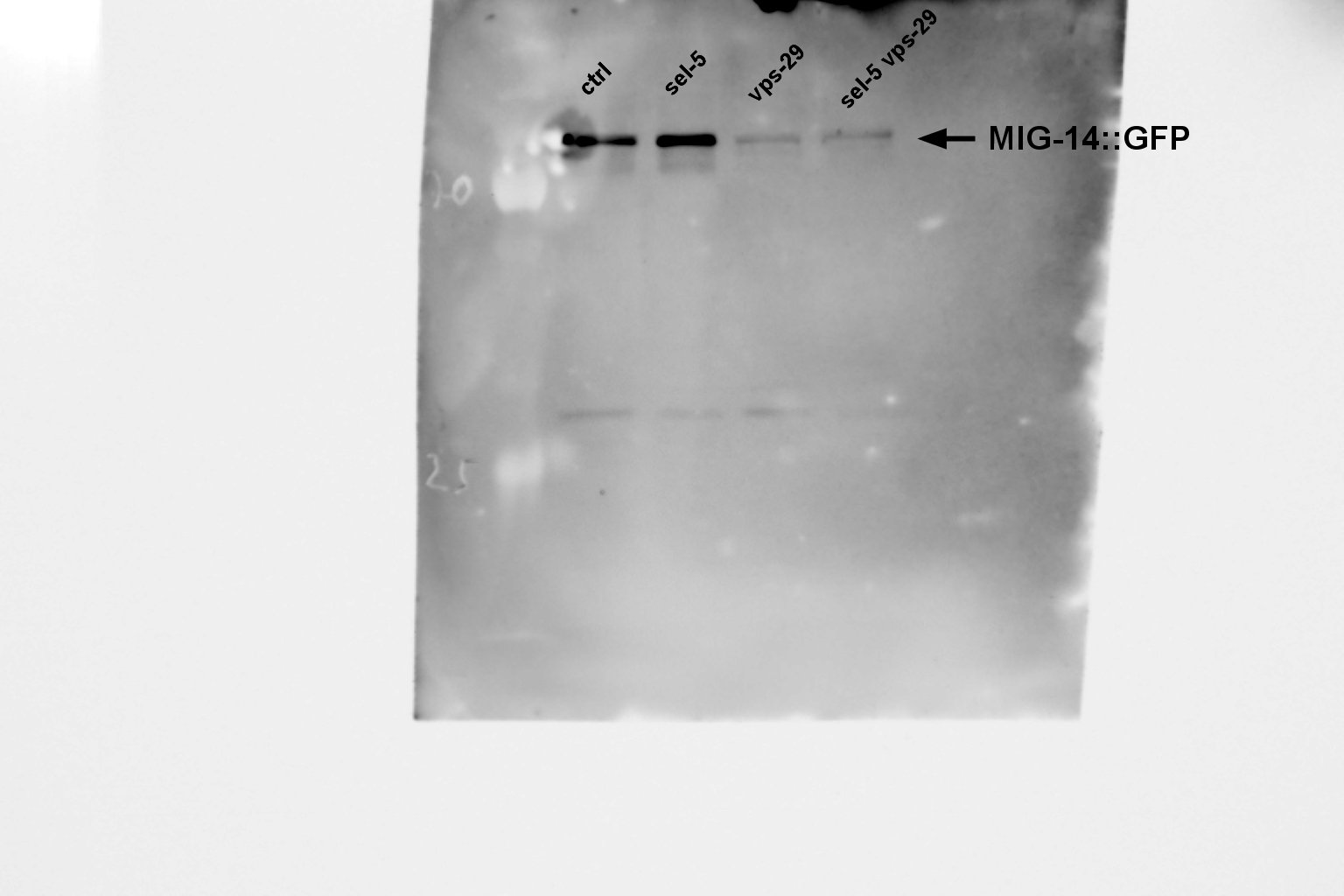

Supplement: Figure 3—source data 1. [file elife-91054-fig3-data1.zip › FIG3D_WB_Sourcedata/FIG3D_04_MIG14GFP_labelled.jpg]

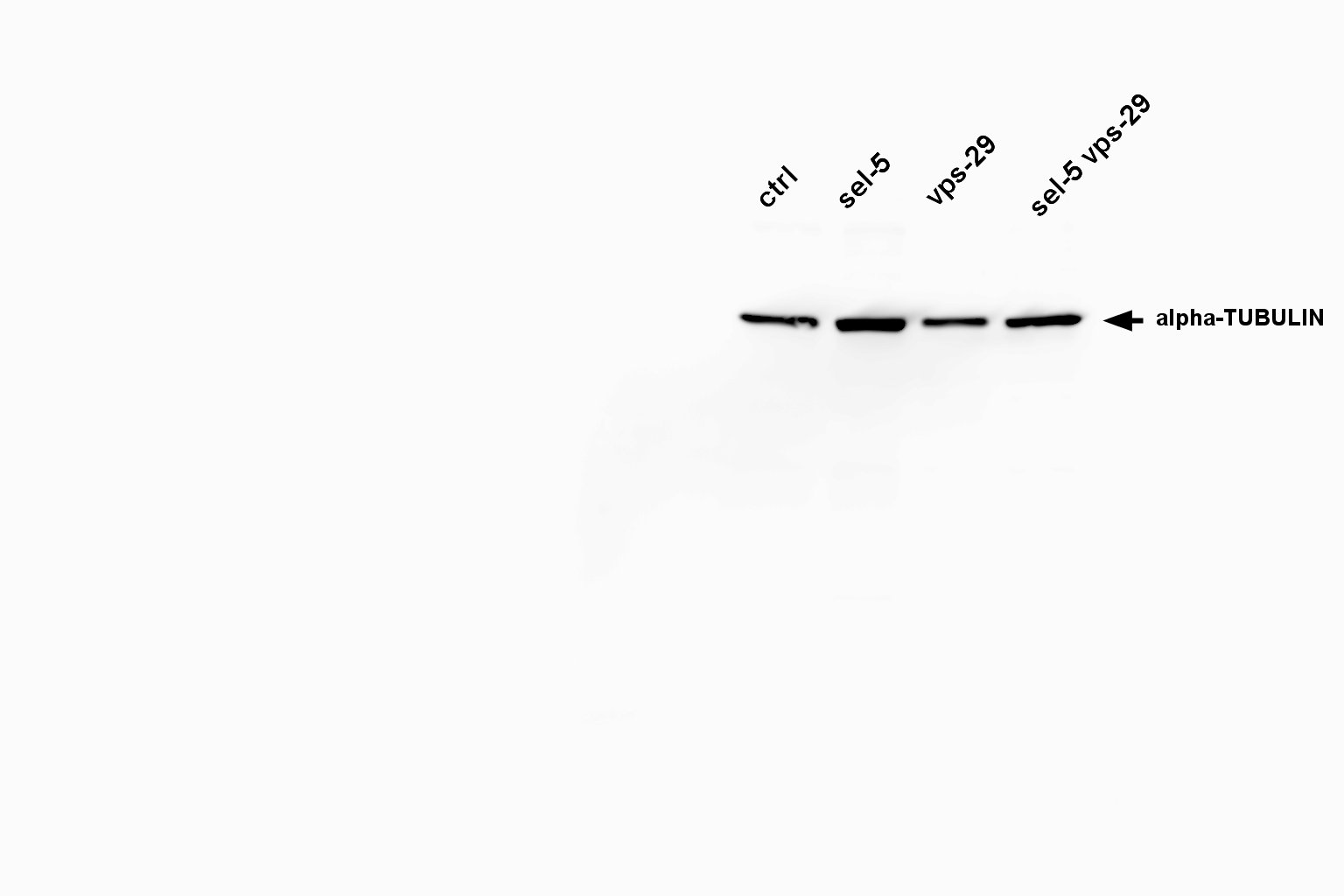

Supplement: Figure 3—source data 1. [file elife-91054-fig3-data1.zip › FIG3D_WB_Sourcedata/FIG3D_05_aTUB_labelled.jpg]

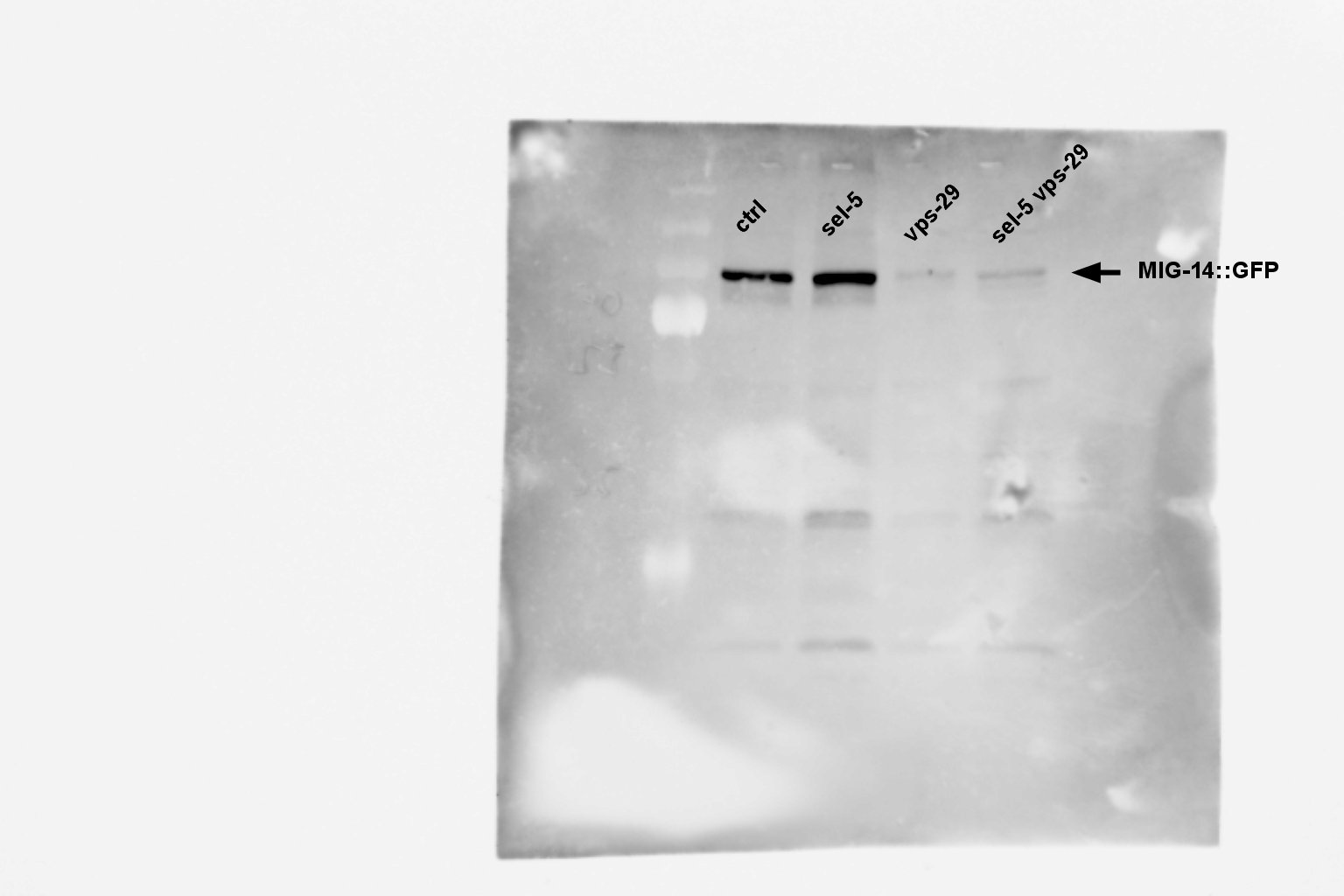

Supplement: Figure 3—source data 1. [file elife-91054-fig3-data1.zip › FIG3D_WB_Sourcedata/FIG3D_05_MIG14GFP_labelled.jpg]

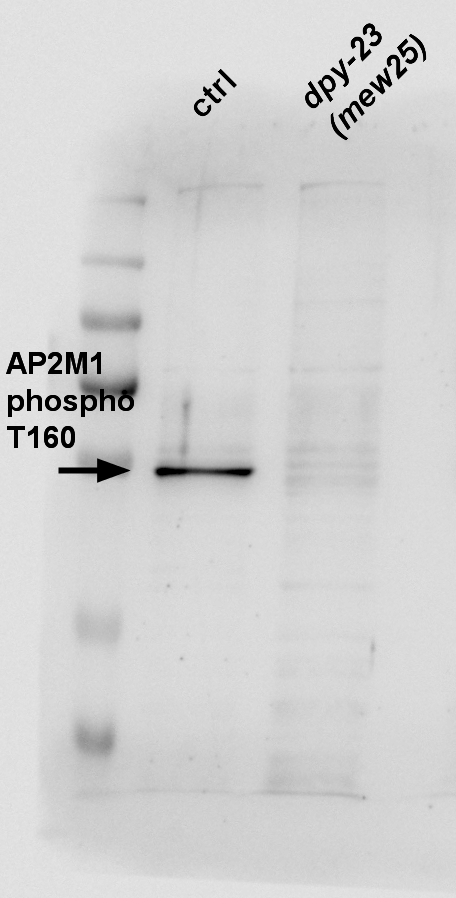

Supplement: Figure 5—source data 1. [file elife-91054-fig5-data1.zip › FIG4B_WB_Sourcedata/Fig4B_01_AP2M1PhosphoT160_labelled.tif]

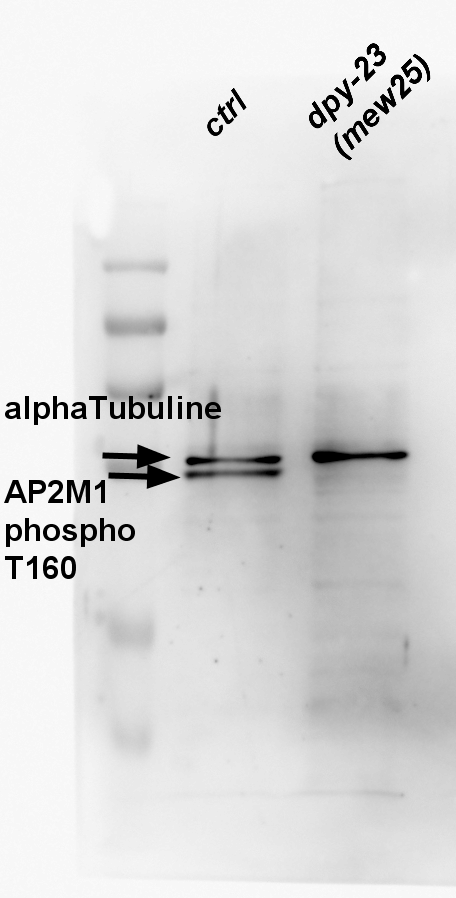

Supplement: Figure 5—source data 1. [file elife-91054-fig5-data1.zip › FIG4B_WB_Sourcedata/Fig4B_01_aTUB_labelled.tif]

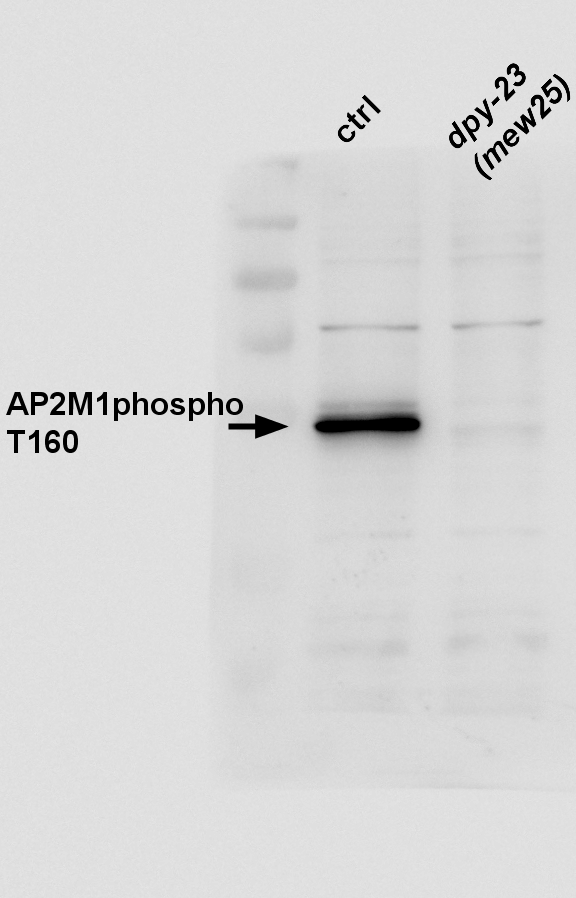

Supplement: Figure 5—source data 1. [file elife-91054-fig5-data1.zip › FIG4B_WB_Sourcedata/Fig4B_02_AP2M1PhosphoT160_labelled.tif]

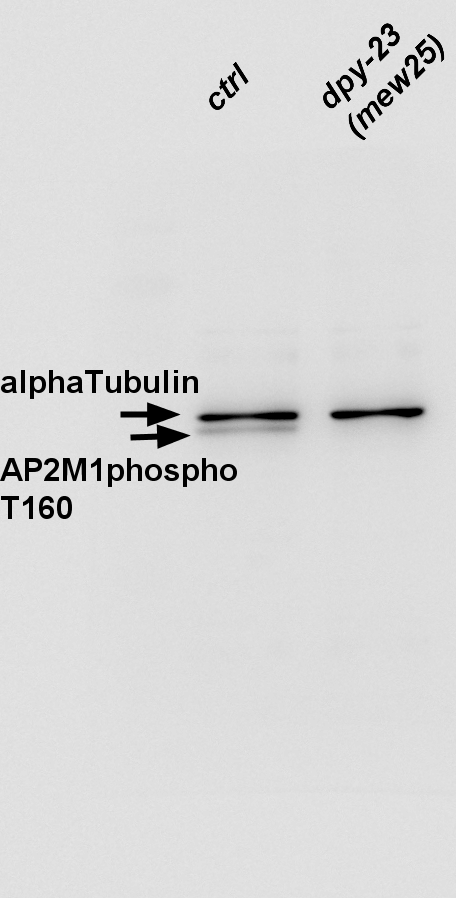

Supplement: Figure 5—source data 1. [file elife-91054-fig5-data1.zip › FIG4B_WB_Sourcedata/Fig4B_02_aTUB_labelled.tif]

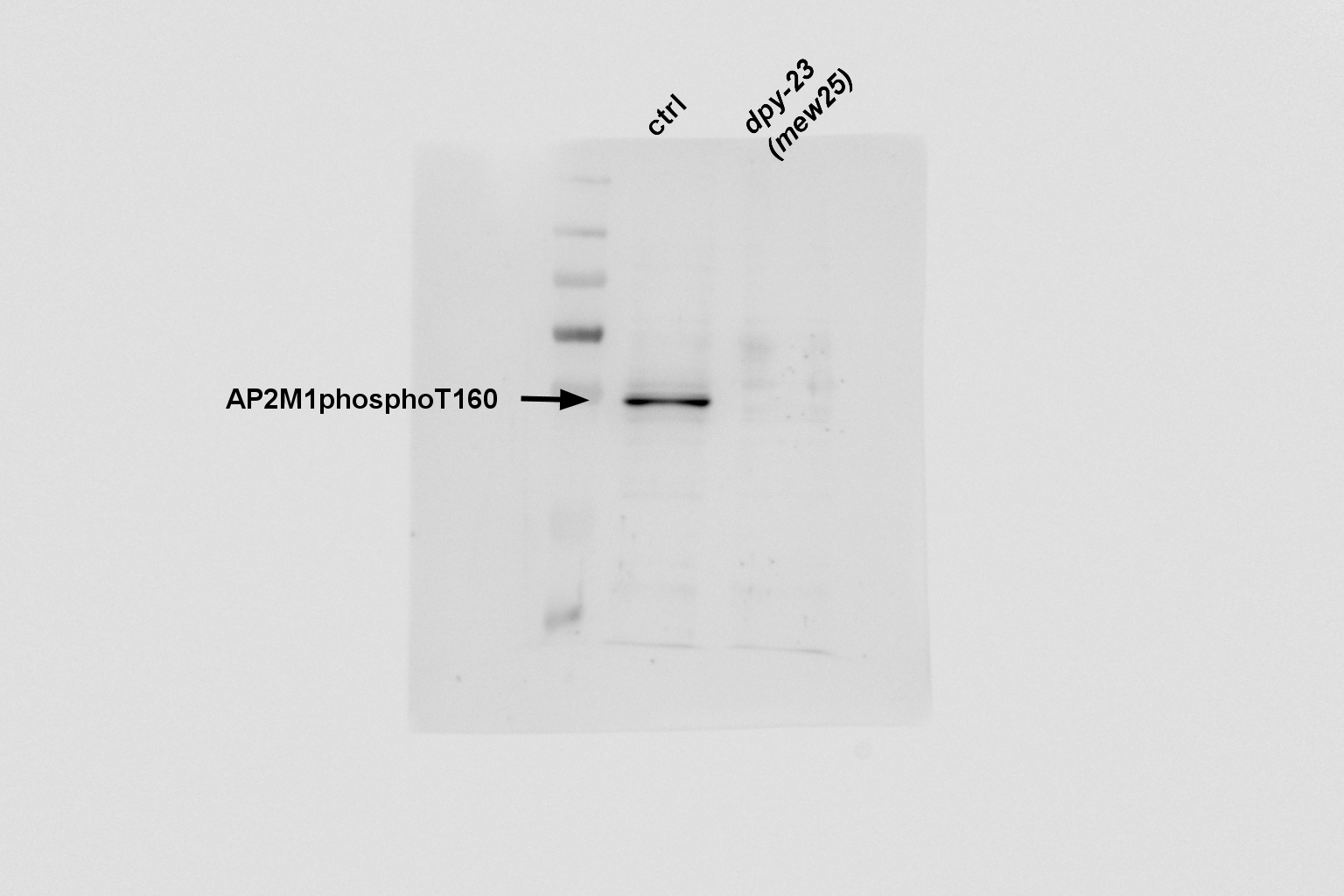

Supplement: Figure 5—source data 1. [file elife-91054-fig5-data1.zip › FIG4B_WB_Sourcedata/Fig4B_03_AP2M1PhosphoT160_labelled.tif]

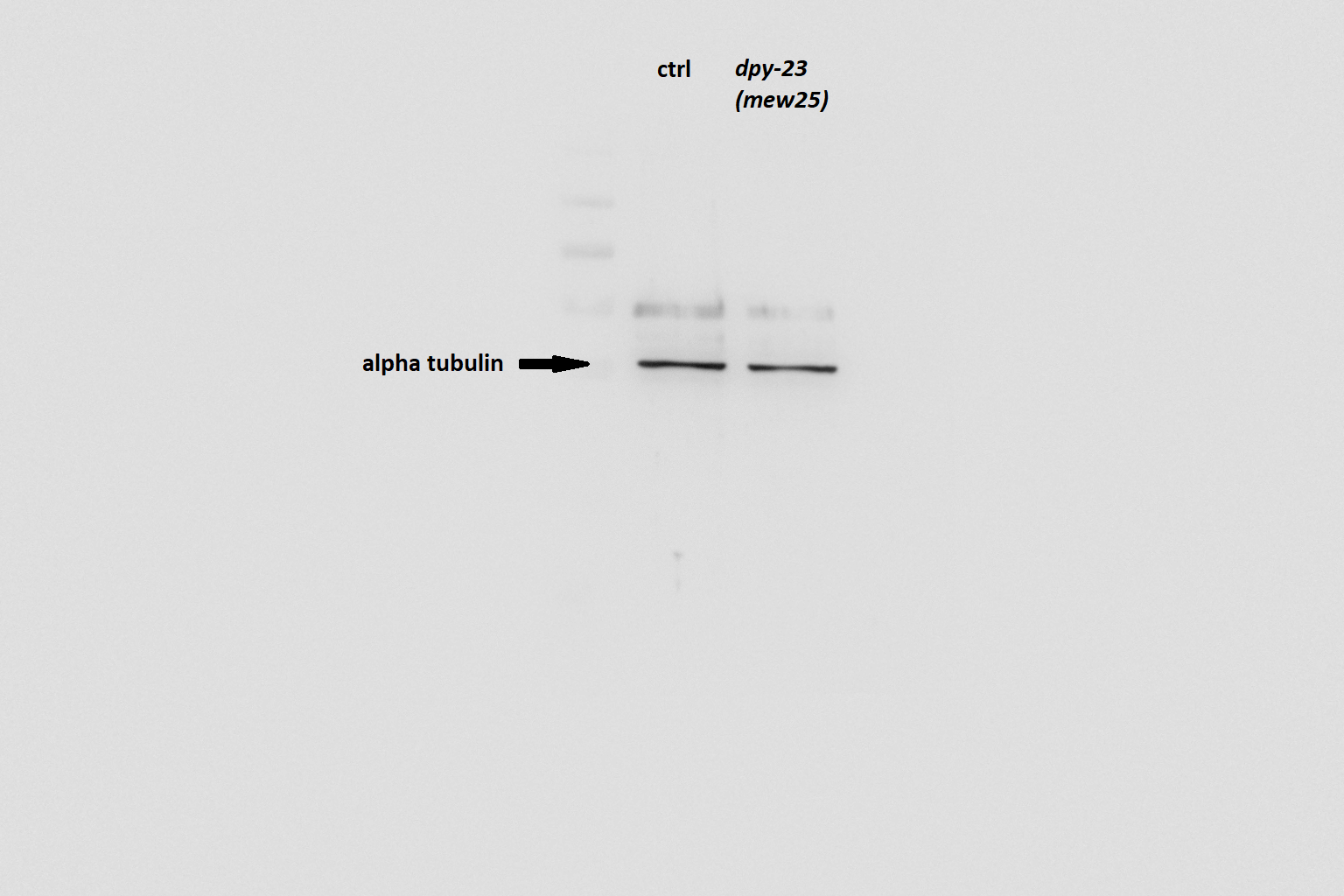

Supplement: Figure 5—source data 1. [file elife-91054-fig5-data1.zip › FIG4B_WB_Sourcedata/Fig4B_03_aTUB_labelled.tif]
